# 

**Published:** 1877-06

**Authors:** 


### Table of Contents.

### ORIGINAL COMMUNICATIONS.

THE WATER SUPPLY OF AUGUSTA. THE NECESSITY FOR ITS PROTECTION AGAINST FURTHER AND FUTURE CONTAMINATION. *By Wm. H. Doughty, M.D., Augusta, Ga.* ........ 129
THE NEW CHEMICAL NOMENCLATURE. *By J. H. Logan, A.M., M.D., Atlanta, Ga.* ........ 147
CINCHONA ALKALOIDS AND THEIR SALTS. *By Ferdinand King, M.D. Ph.G., Atlanta, Ga.* ........ 155

### REPORTS OF SOCIETIES.

Atlanta Academy of Medicine ........ 159

### SELECTIONS.

A Contribution to Medical Jurisprudence ........ 172
Cases Illustrating the Difficulties in Diagnosis and Treatment of Aneurisms of the Lower Extremity ........ 179

### EDITORIAL.

Physic for Uterine Disease ........ 186
American Medical Association ........ 187
American Medical College Association ........ 189
American Dermatological Association ........ 189
Maryland Medical Journal ........ 189
Editorial Correspondence ........ 190, 191

### BIBLIOGRAPHICAL.

The Cure of Rupture, Reducible and Irreducible; also of Variocele and Hydrocele, by New Methods ........ 192
Pamphlets Received ........ 192

